# 3-(Piperidin-1-ium-1-yl)-6-azoniaspiro­[5.5]undecane dibromide monohydrate

**DOI:** 10.1107/S1600536811008713

**Published:** 2011-05-07

**Authors:** Jorge Gonzalez, Roberto Atilano-Coral, Ana Lilia Peraza-Campos, David Ortegón-Reyna, Eleuterio Álvarez

**Affiliations:** a Facultad de Ciencias Químicas, Campus Coquimatlán, Kilometro 9, Carretera Colima-Coquimatlán, Colima, CP 28400, Mexico; b Instituto de Investigaciones Químicas - CSIC - Universidad de Sevilla, Avda. Américo Vespucio 49, 41092 Sevilla, Spain

## Abstract

The title compound, C_15_H_30_N_2_^2+^·2Br^−^·H_2_O, was synthesized by reaction of 4-piperidino­piperidine with dibromo­pentane. The dication is built up from three linked piperidine rings, two of which have one quaternary N atom in common (azoniaspiro), whereas the third is N—C bonded to the azoniaspiro system and protonated on the N atom (piperidinium). All three piperidine rings adopt chair conformations. The crystal structure features O—H⋯Br and N—H⋯Br hydrogen bonds.

## Related literature

For applications of spiro compounds, see: Camblor *et al.* (2001 ▶); Jiang *et al.* (1998 ▶); Kolocouris *et al.* (2007 ▶); Pinto *et al.* (1992 ▶); Salbeck *et al.* (2002 ▶). For related structures, see: Clemente (2003 ▶); Day *et al.* (2005 ▶); Estienne *et al.* (1984 ▶); Huber (1969 ▶); Monkowius *et al.* (2004 ▶); Rosen & Guarino (1991 ▶). For the synthesis, see: Tchoubar & Verrier (1960 ▶).
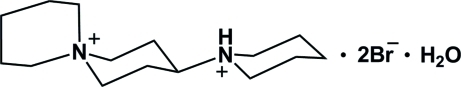


## Experimental

### 

#### Crystal data

C_15_H_30_N_2_^2+^·2Br^−^·H_2_O
*M* *_r_* = 416.25
Monoclinic, 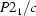

*a* = 6.5491 (2) Å
*b* = 23.3325 (9) Å
*c* = 12.2715 (5) Å
β = 102.141 (1)°
*V* = 1833.23 (12) Å^3^
*Z* = 4
Mo *K*α radiation
μ = 4.42 mm^−1^
*T* = 173 K
0.34 × 0.32 × 0.30 mm

#### Data collection

Bruker–Nonius X8 Kappa APEXII CCD area-detector diffractometer
Absorption correction: multi-scan (*SADABS*; Bruker, 2001 ▶) *T*_min_ = 0.245, *T*_max_ = 0.271
28905 measured reflections
5130 independent reflections
4273 reflections with *I* > 2σ(*I*)
*R*_int_ = 0.026

#### Refinement

*R*[*F* ^2^ > 2σ(*F* ^2^)] = 0.027
*wR*(*F* ^2^) = 0.068
*S* = 1.03
5130 reflections
190 parameters
4 restraints
H atoms treated by a mixture of independent and constrained refinement
Δρ_max_ = 0.81 e Å^−3^
Δρ_min_ = −0.48 e Å^−3^

### 

Data collection: *APEX2* (Bruker, 2007 ▶); cell refinement: *SAINT* (Bruker, 2007 ▶); data reduction: *SAINT*; program(s) used to solve structure: *SIR2002* (Burla *et al.*, 2003 ▶); program(s) used to refine structure: *SHELXL97* (Sheldrick, 2008 ▶); molecular graphics: *SHELXTL* (Sheldrick, 2008 ▶); software used to prepare material for publication: *SHELXTL* and *publCIF* (Westrip, 2010 ▶).

## Supplementary Material

Crystal structure: contains datablocks I, global. DOI: 10.1107/S1600536811008713/qk2002sup1.cif

Structure factors: contains datablocks I. DOI: 10.1107/S1600536811008713/qk2002Isup2.hkl

Additional supplementary materials:  crystallographic information; 3D view; checkCIF report

## Figures and Tables

**Table 1:** Hydrogen-bond geometry (Å, °)

*D*—H⋯*A*	*D*—H	H⋯*A*	*D*⋯*A*	*D*—H⋯*A*
N2—H1*N*2⋯Br1	0.90	2.36 (1)	3.2425 (11)	168 (2)
O1—H1*O*1⋯Br2	0.90	2.48 (1)	3.3664 (8)	168 (1)
O1—H2*O*1⋯Br2^i^	0.90	2.54 (1)	3.3528 (7)	151 (2)

Symmetry code: (i) 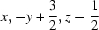.

**Fig. 1.**
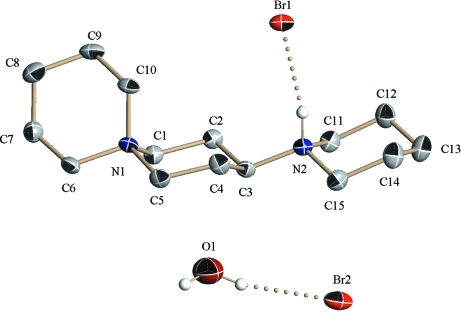
ORTEP drawing of the asymmetric unit of the title compound, (C15H30N2)+2.2(Br-1).H2O, with atom labeling and numbering. Atoms are represented by 50% probability thermal ellipsoids except for H atoms, which are shown as small spheres. The dotted line is a hydrogen bond. C-bonded hydrogen atoms have been omitted for clarity.

**Fig. 2.**
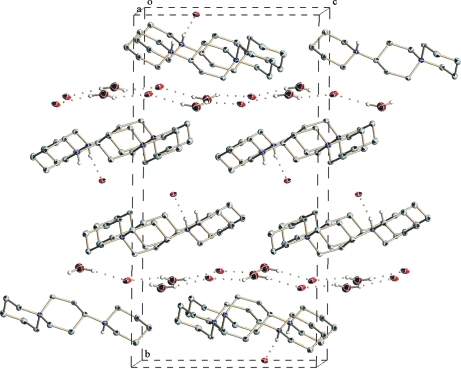
The crystal packing of (I) projected down the a axis. The hydrogen bonds are depicted as dotted lines. H atoms not involved in hydrogen bonding have been omitted for clarity.

## supplementary crystallographic information

### Comment

In the past few years, spiro compounds having cyclic structures fused at a central nitrogen atom have received great attention due to their potential applications in medicine (Kolocouris *et al.*, 2007; Monkowius *et al.*, 2004; Pinto *et al.*, 1992; Rosen and Guarino, 1991), catalysis (Jiang *et al.*, 1998), optical materials (Salbeck *et al.*, 2002) and zeolitic solids synthesis (Camblor *et al.*, 2001). The title compound was synthesized by reaction of 4-piperidinopiperidine with dibromopentane (Tchoubar and Verrier, 1960).

The structure of the title compound is shown in Fig. 1, and the geometrical parameters are given in the Supplementary Information and the archived CIF. The compound crystallized in the centrosymmetric space group *P*2_1_/*c* with one dicationic molecule, two bromide anions and one water molecule in the asymmetric unit. The bond lengths and angles in the dicationic molecule are similar to those observed in some azoniaspiro analogues (Clemente, 2003; Day *et al.*, 2005; Estienne *et al.*, 1984; Huber, 1969). In all these compounds quaternary nitrogen centers appear with a very slightly distorted tetrahedral configuration.

One of the two bromide anions, Br1, is N—H···Br hydrogen bonded to a dicationic molecule and embedded in a double layer of the organocations parallel to (010) showing a number of weak C—H···Br interactions with them (Fig. 1). The second bromide, Br2, and the water molecule form infinite hydrogen bonded chains parallel to [001]. These chains are arranged in layers parallel to (010), which are inserted between the double layers of the organocations and Br1 (Fig. 2).

### Experimental

The title compound (I) was synthesized by reaction of 4-piperidinopiperidine with dibromopentane. 3.0 g of 4-piperidinopiperidine (0.0178 mol) and 4.09 g of 1,5-dibromopentane (0.0178 mol) were dissolved in 170 ml of ethanol. The mixture was heated under reflux for 48 h. After that, the reaction mixture was cooled at 5 °C for 48 h. The precipitate thus formed was recovered by filtration, washed with fresh ethanol and dried at 80°C overnight (yield 70%) and then recrystallyzed from absolute ethanol. Crystals suitable for single-crystal X-Ray diffraction analysis were isolated and data collection was performed in order to determine the molecular structure of (I). The melting point, 336–337 °C (accompanied by thermal decomposition: bubbles were observed to develop during melting), was determined in a Barnstead 1201D Electrothermal MEL-TEMP apparatus.

NMR spectra were recorded on a Jeol 500 MHz spectrometer with D_2_O as solvent. Chemical shifts were expressed in p.p.m. relative to TMS (tetramethylsilane) as internal standard. Signals associated with different hydrogen and carbon atoms (Fig.1) where identified by means of COSY, DEPT and HETCOR experiments.^1^H NMR (500 MHz, D_2_O): δ 3.95 and 3.32 (d, and m, 2H_1ax-eq_, 2H_5ax-eq_), 3.61 (m, 1H_3_), 3.54 (t, 2H_11_, 2H_15_), 3.42 (t, 2H_6_, 2H_10_), 2.30 (m, 2H_2_, 2H_4_), 1.95 (m, 2H_9_, 2H_7_), 1.85 (m, 2H_12_, 2H_14_, 2H_8_), 1.73 (m, 2H_13_). ^13^C NMR (500 MHz, D_2_O): δ 65.1 (C_6_, C_10_), 59.9 (C_3_), 57.1 (C_1_, C_5_), 54.5 (C_11_, C_15_), 23.0 (C_12_, C_14_), 20.9 (C_13_), 20.3 (C_2_, C_4_), 19.3 (C_7_, C_9_), 18.9 (C_8_).

### Refinement

The water hydrogen atoms and the piperidinium N–H were located from a difference Fourier map and refined isotropically, with the O–H and N–H distance restrained both to 0.90 Å, *U*_iso_ = 1.5 *U*_eq_ (O or N). The remaining H atoms were positioned geometrically [C–H = 0.99 Å] and were refined using a riding model, with *U*_iso_ = 1.2 *U*_eq_ (C).

### Crystal data

**Table:**

C_15_H_30_N_2_^2+^·2Br^−^·H_2_O	*F*(000) = 856
*M**_r_* = 416.25	*D*_x_ = 1.508 Mg m^−^^3^
Monoclinic, *P*2_1_/*c*	Mo *K*α radiation, λ = 0.71073 Å
Hall symbol: -P 2ybc	Cell parameters from 9946 reflections
*a* = 6.5491 (2) Å	θ = 2.4–30.5°
*b* = 23.3325 (9) Å	µ = 4.42 mm^−^^1^
*c* = 12.2715 (5) Å	*T* = 173 K
β = 102.141 (1)°	Block, colourless
*V* = 1833.23 (12) Å^3^	0.34 × 0.32 × 0.30 mm
*Z* = 4	

### Data collection

**Table:**

Bruker–Nonius X8 Kappa APEXII CCD area-detector diffractometer	5130 independent reflections
Radiation source: fine-focus sealed tube	4273 reflections with *I* > 2σ(*I*)
graphite	*R*_int_ = 0.026
Detector resolution: 8.26 pixels mm^-1^	θ_max_ = 30.5°, θ_min_ = 2.4°
φ and ω scans with narrow frames	*h* = −5→9
Absorption correction: multi-scan (*SADABS*; Bruker, 2001)	*k* = −33→33
*T*_min_ = 0.245, *T*_max_ = 0.271	*l* = −17→17
28905 measured reflections	

### Refinement

**Table:**

Refinement on *F*^2^	Primary atom site location: structure-invariant direct methods
Least-squares matrix: full	Secondary atom site location: difference Fourier map
*R*[*F*^2^ > 2σ(*F*^2^)] = 0.027	Hydrogen site location: inferred from neighbouring sites
*wR*(*F*^2^) = 0.068	H atoms treated by a mixture of independent and constrained refinement
*S* = 1.03	*w* = 1/[σ^2^(*F*_o_^2^) + (0.0323*P*)^2^ + 1.2017*P*] where *P* = (*F*_o_^2^ + 2*F*_c_^2^)/3
5130 reflections	(Δ/σ)_max_ = 0.007
190 parameters	Δρ_max_ = 0.81 e Å^−^^3^
4 restraints	Δρ_min_ = −0.48 e Å^−^^3^

### Special details

**Table:**

Geometry. All e.s.d.'s (except the e.s.d. in the dihedral angle between two l.s. planes) are estimated using the full covariance matrix. The cell e.s.d.'s are taken into account individually in the estimation of e.s.d.'s in distances, angles and torsion angles; correlations between e.s.d.'s in cell parameters are only used when they are defined by crystal symmetry. An approximate (isotropic) treatment of cell e.s.d.'s is used for estimating e.s.d.'s involving l.s. planes.
Refinement. Refinement of *F*^2^ against ALL reflections. The weighted *R*-factor *wR* and goodness of fit *S* are based on *F*^2^, conventional *R*-factors *R* are based on *F*, with *F* set to zero for negative *F*^2^. The threshold expression of *F*^2^ > σ(*F*^2^) is used only for calculating *R*-factors(gt) *etc*. and is not relevant to the choice of reflections for refinement. *R*-factors based on *F*^2^ are statistically about twice as large as those based on *F*, and *R*- factors based on ALL data will be even larger.

### Fractional atomic coordinates and isotropic or equivalent isotropic displacement parameters (Å^2^)

**Table:**

	*x*	*y*	*z*	*U*_iso_*/*U*_eq_	
Br1	−0.24994 (3)	0.514171 (7)	0.206959 (17)	0.02573 (6)	
N1	0.0568 (2)	0.62958 (6)	−0.05069 (13)	0.0193 (3)	
N2	0.11027 (8)	0.60709 (6)	0.30473 (13)	0.0200 (3)	
H1N2	0.015 (3)	0.5790 (7)	0.2883 (19)	0.030*	
C1	−0.0033 (3)	0.67931 (7)	0.01514 (16)	0.0215 (3)	
H1A	−0.1333	0.6969	−0.0276	0.026*	
H1B	0.1081	0.7087	0.0246	0.026*	
C2	−0.0370 (3)	0.66163 (7)	0.12916 (16)	0.0218 (3)	
H2A	−0.1569	0.6349	0.1199	0.026*	
H2B	−0.0714	0.6959	0.1692	0.026*	
C3	0.1568 (3)	0.63259 (7)	0.19865 (16)	0.0204 (3)	
H3	0.2700	0.6618	0.2192	0.024*	
C4	0.2337 (3)	0.58485 (8)	0.13157 (17)	0.0252 (4)	
H4A	0.3706	0.5708	0.1733	0.030*	
H4B	0.1340	0.5524	0.1231	0.030*	
C5	0.2562 (3)	0.60481 (8)	0.01682 (17)	0.0244 (4)	
H5A	0.3675	0.6342	0.0254	0.029*	
H5B	0.2996	0.5720	−0.0241	0.029*	
C6	0.1035 (3)	0.65089 (8)	−0.16009 (16)	0.0240 (4)	
H6A	0.2073	0.6823	−0.1443	0.029*	
H6B	0.1663	0.6193	−0.1957	0.029*	
C7	−0.0892 (3)	0.67244 (8)	−0.24058 (17)	0.0283 (4)	
H7A	−0.0508	0.6843	−0.3111	0.034*	
H7B	−0.1453	0.7064	−0.2083	0.034*	
C8	−0.2572 (3)	0.62608 (9)	−0.26500 (19)	0.0318 (4)	
H8A	−0.3850	0.6418	−0.3134	0.038*	
H8B	−0.2073	0.5937	−0.3047	0.038*	
C9	−0.3071 (3)	0.60483 (9)	−0.15585 (19)	0.0297 (4)	
H9A	−0.3705	0.6364	−0.1203	0.036*	
H9B	−0.4098	0.5732	−0.1717	0.036*	
C10	−0.1114 (3)	0.58381 (7)	−0.07634 (17)	0.0228 (4)	
H10A	−0.1485	0.5713	−0.0059	0.027*	
H10B	−0.0553	0.5502	−0.1096	0.027*	
C11	0.0009 (3)	0.64804 (8)	0.36873 (17)	0.0251 (4)	
H11A	−0.1286	0.6623	0.3195	0.030*	
H11B	0.0925	0.6813	0.3938	0.030*	
C12	−0.0526 (3)	0.61806 (9)	0.46940 (17)	0.0305 (4)	
H12A	−0.1230	0.6455	0.5109	0.037*	
H12B	−0.1508	0.5862	0.4437	0.037*	
C13	0.1417 (4)	0.59460 (10)	0.54675 (18)	0.0339 (5)	
H13A	0.2347	0.6266	0.5785	0.041*	
H13B	0.1018	0.5734	0.6090	0.041*	
C14	0.2555 (4)	0.55475 (9)	0.48168 (18)	0.0327 (4)	
H14A	0.1677	0.5206	0.4579	0.039*	
H14B	0.3869	0.5416	0.5308	0.039*	
C15	0.3059 (3)	0.58378 (8)	0.37943 (17)	0.0262 (4)	
H15A	0.4057	0.6155	0.4033	0.031*	
H15B	0.3728	0.5558	0.3374	0.031*	
Br2	0.52616 (3)	0.736064 (9)	0.44763 (2)	0.03739 (7)	
O1	0.32909 (7)	0.76797 (9)	0.17852 (6)	0.0581 (5)	
H1O1	0.3996 (2)	0.7627 (12)	0.2491 (4)	0.087*	
H2O1	0.412 (3)	0.7572 (16)	0.1321 (8)	0.087*	

### Atomic displacement parameters (Å^2^)

**Table:**

	*U*^11^	*U*^22^	*U*^33^	*U*^12^	*U*^13^	*U*^23^
Br1	0.01828 (9)	0.02090 (8)	0.03781 (11)	−0.00131 (6)	0.00543 (8)	−0.00437 (7)
N1	0.0162 (7)	0.0175 (6)	0.0255 (8)	−0.0007 (5)	0.0070 (6)	−0.0009 (5)
N2	0.0181 (7)	0.0169 (6)	0.0245 (8)	0.0012 (5)	0.0035 (6)	−0.0005 (5)
C1	0.0231 (8)	0.0161 (7)	0.0254 (9)	0.0024 (6)	0.0052 (7)	−0.0009 (6)
C2	0.0217 (8)	0.0179 (7)	0.0259 (9)	0.0053 (6)	0.0049 (8)	−0.0005 (6)
C3	0.0168 (8)	0.0196 (7)	0.0250 (9)	0.0005 (6)	0.0049 (7)	0.0011 (6)
C4	0.0209 (9)	0.0258 (8)	0.0303 (10)	0.0086 (7)	0.0084 (8)	0.0028 (7)
C5	0.0163 (8)	0.0291 (9)	0.0288 (10)	0.0051 (7)	0.0072 (8)	0.0018 (7)
C6	0.0215 (9)	0.0269 (8)	0.0255 (10)	−0.0042 (7)	0.0097 (8)	0.0004 (7)
C7	0.0301 (10)	0.0292 (9)	0.0259 (10)	−0.0001 (7)	0.0063 (9)	0.0008 (7)
C8	0.0243 (10)	0.0367 (10)	0.0322 (11)	0.0000 (8)	0.0008 (9)	−0.0064 (8)
C9	0.0184 (8)	0.0306 (9)	0.0410 (12)	−0.0058 (7)	0.0082 (9)	−0.0079 (8)
C10	0.0204 (8)	0.0174 (7)	0.0327 (10)	−0.0047 (6)	0.0101 (8)	−0.0042 (7)
C11	0.0254 (9)	0.0226 (8)	0.0272 (10)	0.0069 (7)	0.0054 (8)	−0.0019 (7)
C12	0.0320 (11)	0.0352 (10)	0.0262 (10)	0.0078 (8)	0.0102 (9)	−0.0002 (8)
C13	0.0384 (12)	0.0371 (11)	0.0250 (10)	0.0088 (9)	0.0038 (9)	−0.0001 (8)
C14	0.0378 (11)	0.0297 (9)	0.0296 (11)	0.0102 (8)	0.0045 (10)	0.0046 (8)
C15	0.0220 (9)	0.0257 (9)	0.0290 (10)	0.0068 (7)	0.0013 (8)	0.0026 (7)
Br2	0.02488 (10)	0.02888 (10)	0.05666 (16)	−0.00384 (7)	0.00461 (10)	0.00798 (9)
O1	0.0556 (12)	0.0605 (12)	0.0594 (13)	−0.0124 (10)	0.0147 (11)	−0.0090 (10)

### Geometric parameters (Å, °)

**Table:**

N1—C5	1.507 (2)	C7—H7B	0.9900
N1—C1	1.513 (2)	C8—C9	1.527 (3)
N1—C10	1.519 (2)	C8—H8A	0.9900
N1—C6	1.522 (2)	C8—H8B	0.9900
N2—C11	1.510 (2)	C9—C10	1.520 (3)
N2—C15	1.511 (2)	C9—H9A	0.9900
N2—C3	1.519 (2)	C9—H9B	0.9900
N2—H1N2	0.90000 (14)	C10—H10A	0.9900
C1—C2	1.519 (3)	C10—H10B	0.9900
C1—H1A	0.9900	C11—C12	1.523 (3)
C1—H1B	0.9900	C11—H11A	0.9900
C2—C3	1.530 (2)	C11—H11B	0.9900
C2—H2A	0.9900	C12—C13	1.521 (3)
C2—H2B	0.9900	C12—H12A	0.9900
C3—C4	1.532 (2)	C12—H12B	0.9900
C3—H3	1.0000	C13—C14	1.519 (3)
C4—C5	1.519 (3)	C13—H13A	0.9900
C4—H4A	0.9900	C13—H13B	0.9900
C4—H4B	0.9900	C14—C15	1.522 (3)
C5—H5A	0.9900	C14—H14A	0.9900
C5—H5B	0.9900	C14—H14B	0.9900
C6—C7	1.515 (3)	C15—H15A	0.9900
C6—H6A	0.9900	C15—H15B	0.9900
C6—H6B	0.9900	O1—H1O1	0.90000 (11)
C7—C8	1.527 (3)	O1—H2O1	0.9000
C7—H7A	0.9900		
			
C5—N1—C1	107.06 (14)	C8—C7—H7B	109.4
C5—N1—C10	110.54 (13)	H7A—C7—H7B	108.0
C1—N1—C10	113.04 (13)	C9—C8—C7	109.64 (17)
C5—N1—C6	107.37 (13)	C9—C8—H8A	109.7
C1—N1—C6	110.11 (13)	C7—C8—H8A	109.7
C10—N1—C6	108.57 (14)	C9—C8—H8B	109.7
C11—N2—C15	110.28 (15)	C7—C8—H8B	109.7
C11—N2—C3	113.61 (13)	H8A—C8—H8B	108.2
C15—N2—C3	111.31 (11)	C10—C9—C8	111.16 (16)
C11—N2—H1N2	101.2 (16)	C10—C9—H9A	109.4
C15—N2—H1N2	109.5 (15)	C8—C9—H9A	109.4
C3—N2—H1N2	110.4 (15)	C10—C9—H9B	109.4
N1—C1—C2	112.84 (13)	C8—C9—H9B	109.4
N1—C1—H1A	109.0	H9A—C9—H9B	108.0
C2—C1—H1A	109.0	N1—C10—C9	112.51 (14)
N1—C1—H1B	109.0	N1—C10—H10A	109.1
C2—C1—H1B	109.0	C9—C10—H10A	109.1
H1A—C1—H1B	107.8	N1—C10—H10B	109.1
C1—C2—C3	111.69 (15)	C9—C10—H10B	109.1
C1—C2—H2A	109.3	H10A—C10—H10B	107.8
C3—C2—H2A	109.3	N2—C11—C12	110.28 (15)
C1—C2—H2B	109.3	N2—C11—H11A	109.6
C3—C2—H2B	109.3	C12—C11—H11A	109.6
H2A—C2—H2B	107.9	N2—C11—H11B	109.6
N2—C3—C2	111.00 (13)	C12—C11—H11B	109.6
N2—C3—C4	108.88 (13)	H11A—C11—H11B	108.1
C2—C3—C4	110.52 (15)	C13—C12—C11	111.48 (17)
N2—C3—H3	108.8	C13—C12—H12A	109.3
C2—C3—H3	108.8	C11—C12—H12A	109.3
C4—C3—H3	108.8	C13—C12—H12B	109.3
C5—C4—C3	112.45 (15)	C11—C12—H12B	109.3
C5—C4—H4A	109.1	H12A—C12—H12B	108.0
C3—C4—H4A	109.1	C14—C13—C12	109.32 (18)
C5—C4—H4B	109.1	C14—C13—H13A	109.8
C3—C4—H4B	109.1	C12—C13—H13A	109.8
H4A—C4—H4B	107.8	C14—C13—H13B	109.8
N1—C5—C4	112.68 (14)	C12—C13—H13B	109.8
N1—C5—H5A	109.1	H13A—C13—H13B	108.3
C4—C5—H5A	109.1	C13—C14—C15	112.11 (17)
N1—C5—H5B	109.1	C13—C14—H14A	109.2
C4—C5—H5B	109.1	C15—C14—H14A	109.2
H5A—C5—H5B	107.8	C13—C14—H14B	109.2
C7—C6—N1	112.89 (15)	C15—C14—H14B	109.2
C7—C6—H6A	109.0	H14A—C14—H14B	107.9
N1—C6—H6A	109.0	N2—C15—C14	110.93 (16)
C7—C6—H6B	109.0	N2—C15—H15A	109.5
N1—C6—H6B	109.0	C14—C15—H15A	109.5
H6A—C6—H6B	107.8	N2—C15—H15B	109.5
C6—C7—C8	111.08 (16)	C14—C15—H15B	109.5
C6—C7—H7A	109.4	H15A—C15—H15B	108.0
C8—C7—H7A	109.4	H1O1—O1—H2O1	108.409 (8)
C6—C7—H7B	109.4		
			
C5—N1—C1—C2	−59.45 (18)	C1—N1—C6—C7	−68.66 (19)
C10—N1—C1—C2	62.5 (2)	C10—N1—C6—C7	55.57 (19)
C6—N1—C1—C2	−175.88 (15)	N1—C6—C7—C8	−57.0 (2)
N1—C1—C2—C3	57.2 (2)	C6—C7—C8—C9	55.5 (2)
C11—N2—C3—C2	−48.88 (17)	C7—C8—C9—C10	−55.8 (2)
C15—N2—C3—C2	−174.09 (14)	C5—N1—C10—C9	−173.12 (15)
C11—N2—C3—C4	−170.75 (14)	C1—N1—C10—C9	66.9 (2)
C15—N2—C3—C4	64.03 (18)	C6—N1—C10—C9	−55.58 (19)
C1—C2—C3—N2	−171.29 (13)	C8—C9—C10—N1	57.4 (2)
C1—C2—C3—C4	−50.38 (19)	C15—N2—C11—C12	−57.94 (19)
N2—C3—C4—C5	172.25 (14)	C3—N2—C11—C12	176.30 (15)
C2—C3—C4—C5	50.1 (2)	N2—C11—C12—C13	58.5 (2)
C1—N1—C5—C4	58.59 (19)	C11—C12—C13—C14	−56.2 (2)
C10—N1—C5—C4	−64.92 (19)	C12—C13—C14—C15	55.0 (3)
C6—N1—C5—C4	176.81 (15)	C11—N2—C15—C14	56.78 (19)
C3—C4—C5—N1	−56.1 (2)	C3—N2—C15—C14	−176.17 (15)
C5—N1—C6—C7	175.11 (15)	C13—C14—C15—N2	−56.1 (2)

### Hydrogen-bond geometry (Å, °)

**Table:**

*D*—H···*A*	*D*—H	H···*A*	*D*···*A*	*D*—H···*A*
N2—H1N2···Br1	0.90	2.36 (1)	3.2425 (11)	168 (2)
O1—H1O1···Br2	0.90	2.48 (1)	3.3664 (8)	168.(1)
O1—H2O1···Br2^i^	0.90	2.54 (1)	3.3528 (7)	151.(2)

Symmetry codes: (i) *x*, −*y*+3/2, *z*−1/2.
